# Comparing Hormone Therapies in Peri- and Post-Menopausal Women With Psychosis: A Literature Review

**DOI:** 10.1192/bjo.2025.10796

**Published:** 2025-06-20

**Authors:** Catherine Kennedy, Noëmie Praud

**Affiliations:** McMaster University, Hamilton, Canada

## Abstract

**Aims:** The menopausal transition, marked by a sharp decline in oestrogen, is increasingly recognised as a critical period for new onset of psychosis or worsening symptoms in women with pre-existing schizophrenia, yet treatment strategies remain largely overlooked. Oestrogen’s profound neuroprotective effects – modulating dopaminergic, serotonergic, and glutamatergic pathways, and mediating neural apoptosis – suggest that hormone-based therapies could revolutionize the management of menopause-associated psychosis (MAP) and exacerbation of pre-existing schizophrenia in peri- and post-menopausal women. However, concerns over the long-term risks of hormone replacement therapy (HRT), including breast and uterine cancer and cardiovascular disease, have hindered its widespread adoption. In contrast, the emergence of selective oestrogen receptor modulators (SERMs) have offered a novel, safer alternative with a potentially broader therapeutic window. This review synthesises the current evidence, explores the differential efficacy of hormone therapies including HRT and SERMs, compares response between the above populations of peri- and post-menopausal women with psychosis, and identifies gaps in the literature that warrant further investigation. To our knowledge, this is the first literature review specifically comparing efficacy of HRT vs SERMs in the peri- and post-menopausal population of women with psychosis.

**Methods:** To address the questions posed about (1) efficacy of SERMs vs HRT in (2) menopause-associated psychosis vs menopausal women with pre-existing schizophrenia, the authors searched PubMed databases from years 1990 to 2025, with various combinations of the following terms: schizophrenia, late-onset schizophrenia, psychosis, late-onset psychosis, menopause, perimenopause, postmenopause, menopause-associated psychosis, oestrogen, estradiol, raloxifene, HRT, and SERMs. Over 743 relevant abstracts were found, and narrowed down to 72 reviews and experimental studies to be included in this review. Studies were selected based on their applicability to answer the authors’ questions.

**Results:** This comprehensive review reveals that both HRT and SERMs significantly alleviate not only psychotic but also cognitive and negative symptoms, with SERMs demonstrating superior long-term safety and sustained efficacy, as well as a longer therapeutic window of action. Crucially, differences in treatment response between menopause-associated psychosis and pre-existing schizophrenia suggest that hormonal interventions must be tailored to their underlying pathophysiological mechanisms.

**Conclusion:** These findings challenge the current paradigm of psychosis treatment in menopausal women and underscore the urgent need for large-scale, longitudinal studies to refine dosing strategies, assess long-term psychiatric and cognitive outcomes, and explore potential synergies between hormonal and antipsychotic therapies. Expanding research in this area could redefine psychiatric care for midlife women, offering more personalised and effective treatment pathways.

